# Highlighting the genetic and epidemiologic disparities of *Mycobacterium tuberculosis* epidemic in 12 Caribbean territories in a first global study

**Published:** 2011-01-10

**Authors:** Julie Millet, Shirematee Baboolal, Nalin Rastogi

**Affiliations:** 1 Tuberculosis and Mycobacteria Unit, Institut Pasteur de Guadeloupe, Abymes, Guadeloupe; 2 University of the West Indies, St. Augustine Campus, St Augustine, Trinidad and Tobago

## 

Tuberculosis (TB) in the Caribbean remains a significant health issue with many countries exceeding the WHO target of 5 cases / 100,000 populations. As a developing nation, many of these Caribbean countries face serious challenges in the diagnosis, treatment, care and management of patients with tuberculosis. In light of the current problems facing the tuberculosis programs in the Caribbean, there is a need for studies to be conducted so as to better understand the epidemiology of this disease in such a heterogeneous setting. This investigation describes a first global molecular epidemiological study on 480 clinical *M. tuberculosis* isolates from as many patients, collected in 12 territories of the Caribbean: Bahamas, Barbados, Belize, Dominica, Guyana, Jamaica, St. Kitts and Nevis, St. Lucia, St. Vincent and the Grenadines, Suriname, Trinidad and Tobago, Turks and Caicos. Analysis of “de-identified” patient data showed that TB cases more often concerned males (male to female sex-ratio, 3.1), and persons within age group 25-45 years. The rate of TB/HIV coinfection was unexpectedly high with rates ranging from 44.4% in Guyana, 42.9% in Bahamas, 30.6% in Trinidad and Tobago, 21.4% in Suriname, 14.3% in Barbados and 13.5% in Jamaica. The highest rate of drug-resistant TB was observed in Guyana (27.8%, among which 76% were multidrug-resistant). Spoligotyping generated a total of 104 distinct patterns for the 480 isolates studied; 49 patterns containing 425 isolates (88.5%) corresponded to clustered strains (2-93 isolates per cluster), while the remaining 55 patterns corresponded to unclustered strains (11.5%). A comparison of the spoligotypes with the SITVIT2 global database showed that the isolates belonged to the following predominant genotypic lineages: the ill-defined T lineage (31.0%), East-African Indian (EAI, 19.0%), Latin American and Mediterranean (LAM, 10.4%), the X clade (8.3%), Haarlem (5.8%), and Beijing (3.5%). The diversity of strains circulating in the Caribbean essentially represented their colonial past (clades of European descent such as Haarlem, and X clades) as well as population movements (EAI, Beijing). Lineages characteristic of the Indian subcontinent (East- African-Indian, Central-Asian) were seen in Trinidad and Tobago, Guyana, and Suriname where there is a large population of East Indians brought during the indentureship period, after slavery was abolished. Lastly, a peculiar local evolution of *M. tuberculosis* strains in Trinidad and Tobago was evidenced with the exclusive local emergence of a specific TB clone (named SIT566, belonging most probably to the X clade), which resulted in 56% of all TB cases.

